# Real-World Evidence for COVID-19 Delta Variant's Effects on the Digestive System and Protection of Inactivated Vaccines from a Medical Center in Yangzhou, China: A Retrospective Observational Study

**DOI:** 10.1155/2022/7405448

**Published:** 2022-08-19

**Authors:** Wenjing Zhao, Yong Li, Ruijin Xie, Yuying Dong, Yan Wei, Ce Cheng, Scott Lowe, Chenyu Sun, Cunjin Wang, Ju Gao

**Affiliations:** ^1^Affiliated Northern Jiangsu People's Hospital of Yangzhou University, Yangzhou, China; ^2^Affiliated Hospital of Jiangnan University, Wuxi, China; ^3^Center for Disease Control and Prevention, Yangzhou, China; ^4^The University of Arizona College of Medicine, Tucson, ARI, USA; ^5^Kansas City University, College of Osteopathic Medicine, Kansas, MO, USA; ^6^Internal Medicine, AMITA Health Saint Joseph Hospital Chicago, Chicago, IL, USA

## Abstract

**Background:**

Coronavirus disease 2019 (COVID-19) is rapidly disseminated worldwide, and it continues to threaten global public health. Recently, the Delta variant has emerged as the most dreaded variant worldwide. COVID-19 predominantly affects the respiratory tract, and studies have reported the transient effects of COVID-19 on digestive system function. However, the relationship between the severity of the Delta variant and digestive system function remains to be investigated. Additionally, data on the ability of the inactive Chinese vaccines (Sinovac or Sinopharm) to protect against the Delta variant or COVID-19-induced gastrointestinal symptoms in the real world are insufficient. Thus, the present retrospective observational study first attempted to use the total gastrointestinal symptom rating scale scores (GSRS) to quantify the possible changes in digestive system functions following the Delta variant infection in the early stage. In addition, the study discusses the potential of inactivated vaccines in preventing severe or critical symptoms or Delta variant-induced digestive system dysfunction.

**Methods:**

To evaluate the difference between mild illness group, moderate illness group, and severe or critical illness group, analysis of variance (ANOVA) was employed to compare the three groups' total gastrointestinal symptom rating scale scores (GSRS). A chi-squared test was used to compare the differences in the ratio of the abnormal biochemical measurements among the three groups first. Then, the percentage of the vaccinated population was compared among the three groups. Additionally, the ratio of the abnormal serum markers between the vaccinated and nonvaccinated cohorts was compared. A *P* value < 0.05 was considered statistically significant.

**Results:**

Significant differences were observed in the abnormal ratio of alanine aminotransferase (ALT), total bilirubin (TBIL), direct bilirubin (DBIL), lactate dehydrogenase (LDH), and Interleukin 6 (IL-6) ratio among the three groups (*P* < 0.05). Additionally, no significant difference was observed in the abnormal serum markers ratio between day 14 and day 21 after treatment (*P* > 0.05). A significant difference was observed in the total GSRS scores among the three groups and the ratio of the vaccinated population among the three groups (*P* < 0.05). A significant difference was observed in the ratio of the abnormal serum ALT and AST levels between the vaccinated and nonvaccinated cohorts (*P* < 0.05).

**Conclusions:**

In summary, serum AST, DBIL, LDH, and IL-6 levels are potential markers for distinguishing severe or critical patients in the early stage of the Delta variant infection. Additionally, changes in the levels of these serum makers are transient, and the levels can return to normal after treatment. Furthermore, severe gastrointestinal discomfort was significantly more prevalent in patients with severe or critical diseases and should thus be considered in patients diagnosed with Delta variant infection. Finally, inactivated vaccines may prevent severe or critical symptoms and Delta variant-induced liver dysfunction. Vaccination programs must be promoted to protect public health.

## 1. Introduction

Coronavirus disease 2019 (COVID-19) is caused by novel severe acute respiratory syndrome coronavirus-2 (SARS-CoV-2) and was first detected in Wuhan, China, on December 31, 2019 [1]. Since its first report, the disease has rapidly disseminated worldwide, and it continues to threaten global public health [2]. As of December 2, 2021, approximately 262,346,000 confirmed cases and 5,224,116 deaths worldwide had been reported due to the COVID-19 pandemic [3].

Like other RNA viruses, SARS-CoV-2 undergoes mutations over time, with its first variant, Alpha (B.1.1.7), reported in the United Kingdom in December 2020. To date, four variants of the virus, namely, Alpha (B.1.1.7), Beta (B.1.351), Gamma (P.1), and Delta (B.1.617.2), have been identified by the World Health Organization. The Delta variant is 60% more transmissible than the Alpha variant [4]. Thus, the Delta variant has emerged as the most dreaded variant worldwide, and it has been responsible for almost all new SARS-CoV-2 cases in the United States since July 2021 [5].

COVID-19 predominantly affects the respiratory tract, with the most common clinical manifestations being fever, dry cough, fatigue, and myalgia [6]. Previous studies have reported the transient effects of COVID-19 on digestive system functions [7, 8]. However, further investigations are required to ascertain the possible relationship between the severity of the Delta variant and digestive system functions. Additionally, data on the ability of the inactive Chinese vaccines (Sinovac or Sinopharm) to protect against the Delta variant or COVID-19-induced gastrointestinal symptoms in the real world are insufficient.

In August 2021, the Delta variant spread in Yangzhou, China. Thus, the present retrospective observational study first attempted to use the total gastrointestinal symptom rating scale (GSRS) scores to quantify possible changes in the digestive system functions following the Delta variant infection in its early stage. In addition, the study discusses the potential of inactivated vaccines in preventing severe or critical symptoms or Delta variant-induced digestive system dysfunction.

## 2. Materials and Methods

### 2.1. Data Collection

**Figure 1:**
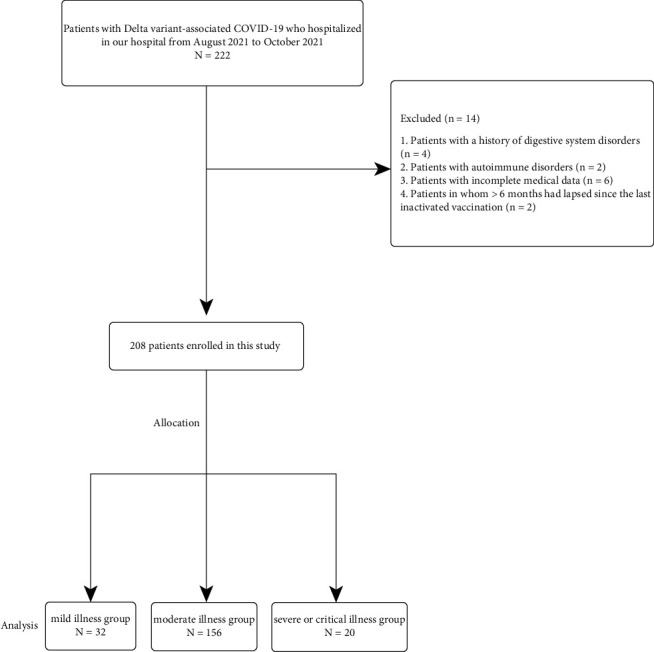
Flow chart of the study protocol.

The present retrospective observational study reviewed the medical records of 208 patients diagnosed with Delta variant-associated COVID-19 through high-throughput whole genome sequencing and hospitalized at the Affiliated Northern Jiangsu People's Hospital of Yangzhou University from August 2021 to October 2021. The patients were divided into three groups, that is, mild illness group, moderate illness group, and severe or critical illness group, according to the Eighth Edition of the Chinese official guidelines for COVID-19 (Supplementary File 1: Eighth Edition of the Chinese official guidelines for COVID-19). Patients with a history of digestive system disorders such as cholangitis and hepatitis, congenital malformations, and gastrointestinal tumors were excluded from the study. Patients with autoimmune diseases such as dermatomyositis, systemic lupus erythematosus, and acquired immunodeficiency syndrome; those with incomplete medical data; and patients in whom >6 months had elapsed since the last inactivated vaccination were also excluded. The workflow used to screen for eligible participants is illustrated in Figure 1.

### 2.2. Ethical Considerations

Written informed consent was acquired from all the patients who participated in this study to obtain anonymous data from the medical records. The Research Ethics Committees of the Affiliated Northern Jiangsu People's Hospital of Yangzhou University (Yangzhou, China) approved the study (approval number: 2021ky284; approval date: 2021/11/18).

### 2.3. Primary Measurements

#### 2.3.1. Biochemical Measurements

Biochemical measurements were performed to investigate the possible correlation between the digestive system function and the severity of the Delta variant. The serum levels of alanine aminotransferase (ALT; normal: 10–40 U/L), aspartate aminotransferase (AST; normal: 8–40 U/L), total bilirubin (TBIL; normal: 3.4–17.1 *μ*mol/L), lactate dehydrogenases (LDH; normal: 100–280 U/L), and direct bilirubin (DBIL; normal: 1.7–10.2 *μ*mol/L) were recorded upon admission [9, 10]. These parameters were re-recorded after 7, 14, and 21 days of treatment to confirm whether these changes were transient. Additionally, the interleukin-6 (IL-6, average < 7 pg/mL) level, which acts as an indicator for several diseases [11], was recorded to explore the possible relationship between the proinflammatory cytokine and Delta variant.

### 2.4. Gastrointestinal Symptom Rating Scale

The Chinese version of the gastrointestinal symptom rating scale (GSRS) was used through “ask and answer” to compare the severity of gastrointestinal symptoms among the three groups [12] (Supplementary File 2: Chinese version of the gastrointestinal symptom rating scale). The English version of the GSRS often uses 7-graded Likert scales; unlike the English version, the Chinese version comprises 15 items and five subscales, namely, dyspepsia, diarrhea, abdominal pain, reflux, and constipation [13, 14]. Each item is scored on a 1–4 Likert scale; the higher the score, the greater the severity of symptoms. The Chinese version of the GSRS has been verified and used extensively for more than ten years to assess gastrointestinal discomfort in Chinese patients [12, 15–17]. The present study recorded GSRS scores on admission and compared the total GSRS score to determine differences in the gastrointestinal symptoms among the three groups.

### 2.5. Ratio of the Vaccinated Population in Three Groups

The present study compared the ratio of the vaccinated population in three groups to investigate the potential of the inactivated vaccines in preventing severe or critical symptoms. In addition, this study also compared the efficiency of single-dose and two-dose inactivated vaccines against the Delta strain infection.

### 2.6. Ratio of the Abnormal Serum Markers in the Vaccinated and Nonvaccinated Cohorts

Differences in the ratio of the abnormal serum ALT, AST, LDH, TBIL, and DBIL levels between the vaccinated and nonvaccinated cohorts were compared to determine the potential of inactivated vaccines in preventing COVID-19-induced digestive system dysfunction.

### 2.7. Statistical Analyses

Statistical analyses were performed using SPSS version 23.0 (IBM Corporation, USA). The results are presented as ratios or means ± standard deviations (SD). Analysis of variance (ANOVA) was employed to compare the total GSRS scores among the three groups. First, the chi-squared test was used to compare differences in the ratio of the abnormal biochemical measurements among the three groups. Then, the percentage of the vaccinated population was compared among the three groups. Additionally, the ratio of the abnormal serum markers between the vaccinated and nonvaccinated cohorts was compared. A *P* value < 0.05 was considered statistically significant.

## 3. Result

### 3.1. Demographics

Patient demographics and laboratory findings are presented in Tables 1 and 2, respectively. Patients' age ranged from 2 to 91 years (mean age: 53.5 years; SD: 20.9 years), and the majority (*n* = 118, 56%) of the patients were women. Of the 208 patients, 83 (39%) patients were more than 60 years old. Moreover, 32 (15.3%) patients reported gastrointestinal discomfort before being diagnosed with the Delta variant infection in the past week. The gastrointestinal symptoms often manifest as diarrhea, nausea, vomiting, or diminished appetite.

### 3.2. Primary Measurements

#### 3.2.1. Biochemical Measurements

Of the 208 patients, 33 (15%) patients exhibited abnormal ALT levels, 44 (21%) patients exhibited abnormal AST levels, 18 (8%) patients exhibited abnormal TBIL levels, 43 (20%) patients exhibited abnormal DBIL levels, 31 (14%) patients exhibited abnormal LDH levels, and 159 (75%) patients exhibited abnormal IL-6 levels. No significant difference was observed in the ratio of abnormal ALT levels (*P* > 0.05). In contrast, significant differences were observed in the abnormal ratio of AST, TBIL, DBIL, LDH, and the IL-6 ratio among the three groups (*P* < 0.05) (Table 3). Multiple comparison results of AST, LDH, and IL-6 levels in the three groups indicated a significant difference in the abnormal serum markers ratio between the patients with mild or moderate illness and those with severe or critical illness (*P* < 0.05; Table 3). No significant difference was observed in the abnormal serum markers ratio between the patients with mild illness and those with moderate illness (*P* > 0.05). However, the abnormal ratio of DBIL differed significantly among all groups, and the TBIL level varied significantly between the mild illness and severe or critical illness groups. Additionally, the level of most serum markers returned to normal within 14 days, and no significant difference was observed in the abnormal serum markers ratio between day 14 and day 21 after treatment (*P* > 0.05, Supplementary File 3).

### 3.3. Gastrointestinal Symptom Rating Scale

The present retrospective study explored the association between the total GSRS scores and three groups in the 32 (15.3%) patients with gastrointestinal discomfort. A significant difference was observed in the total GSRS scores among the three groups (*P* < 0.05; Table 4). Additionally, the difference in the scores between patients with mild or moderate illness and those with severe or critical illness was statistically significant (*P* < 0.01). No significant difference (*P* > 0.05) was observed between the patients with mild illness and those with moderate illness. Although all groups presented with gastrointestinal discomfort, severe discomfort was observed in the patients infected with the Delta variant who exhibited severe or critical illness in the early stage.

### 3.4. Ratio of the Vaccinated Population in Three Groups

A significant difference was observed in the ratio of the vaccinated population among the three groups *P* < 0.05; Table 5). Multiple comparison results indicated a significant difference in the ratio of the vaccinated population between the mild or moderate and severe or critical patients (*P* < 0.05). No significant difference (*P* > 0.05) was observed between the patients with mild illness and those with moderate illness. As expected, the two-dose inactivated vaccine was found to be more efficient in preventing severe or critical symptoms (*P* < 0.05; Table 6). Consistent with previous results, our study indicated that the inactivated vaccines (Sinovac or Sinopharm) might be useful in preventing severe/critical symptoms in infected patients.

### 3.5. Ratio of the Abnormal Serum Markers in the Vaccinated and Nonvaccinated Cohorts

A significant difference was observed in the abnormal serum ALT and AST levels ratio between the vaccinated and nonvaccinated cohorts (*P* < 0.05; Table 7). No significant difference was observed in the ratio of the abnormal serum LDH, TBIL, and DBIL (*P* > 0.05; Table 7). All the patients enrolled in this study had no history of digestive system disorders, and elevated liver transaminase levels may indicate the liver dysfunction among COVID-19 patients [18, 19]. Our study indicated that the inactivated vaccines might prevent Delta variant-induced liver dysfunction.

## 4. Discussion

To the best of our knowledge, this retrospective study from one medical center is the first report regarding the use of GSRS to systematically investigate changes in the digestive system function in the early disease stage and determine the efficiency of inactivated vaccines in preventing severe or critical symptoms in people infected with the Delta variant.

Unlike other cities affected by the COVID-19 pandemic in China, the pandemic outbreak in Yangzhou was initiated in the chess and card room, where elderly people often choose to play mahjong. Poorly ventilated air and crowd accelerated the spread of the virus, which resulted in the COVID-19 incidence in a high proportion of elderly patients (39% of our hospital patients aged >60 years) [20].

SARS-CoV-2 is the seventh coronavirus identified as having human infection capacity by the Chinese authorities [21]. In a study from Wuhan, 2%–10% of the patients diagnosed with COVID-19 exhibited gastrointestinal symptoms such as diarrhea and vomiting [22]. Numerous studies have indicated that COVID-19 also affects the digestive system [23, 24]. SARS-CoV-2 detection in stool specimens and the intestinal autopsies from COVID-19 patients has confirmed SARS-CoV-2's intestinal damage potential [25, 26]. Several clinical studies on COVID-19 have demonstrated liver dysfunction in the affected patients that mainly presents as abnormal ALT and AST levels accompanied by elevated LDH and bilirubin levels [8, 27, 28]. Experts indicated that patients with coronavirus infections might be directly caused by the cytopathic effect of viruses on hepatocytes and cholangiocytes, and an Italian autopsy report confirms the widespread liver vascular injury in the patients without preexisting medical comorbidities [29, 30]. It should not be neglected that drug-induced liver injury might induce the liver dysfunction in patients due to present antiviral drugs or Chinese herbs that may cause direct hepatocyte toxicity [29, 31]. Previous reports from Wuhan report that nearly 56% of patients experienced liver dysfunction after treatment with lopinavir and ritonavir. Drug-induced liver injury may explain the observed broad variability across the different study cohorts [31, 32]. As it is still an ongoing scientific report to use the updated Roussel Uclaf Causality Assessment Method (RUCAM) to define the causality of drug-induced liver injury among COVID-19 patients, it might also be helpful in managing drug-induced liver injury based on the liver injury-specific databases such as the Chinese official database Hepatox, or the US official database LiverTox [31, 33–35]. The results in the present study are congruent with previous reports. Thus, serum AST, DBil, LDH, and IL-6 levels may be potential markers to identify patients with severe or critical infections in the early stage. In contrast, changes in the level of these biomarkers are transient, as reported in many previous studies [36]. Additionally, the Delta variant-induced only transient changes in ALT, AST, DBil, TBil, and LDH levels, all of which returned to normal within 14 days after treatment. Thus, no significant difference was observed in the abnormal serum markers ratio between days 14 and 21. Furthermore, the total GSRS scores identified severe or critical patients in the early stage compared to mild or moderate disease.

The exact mechanism of COVID-19 in inducing digestive system dysfunction is unclear to date [37]. Three hypotheses are available to explain gastrointestinal symptoms in several COVID-19 patients. According to the hypothesis proposed to explain the digestive system dysfunction during COVID-19 infections, the downregulation of angiotensin-converting enzyme 2 (ACE2) expression in the intestinal tract is the primary factor [38]. Previous studies have reported that the ACE2 levels decreased during the SARS-CoV infection, and ACE2 is known for its effect as the main counterregulatory enzyme to ACE that functions by the breakdown of degrading angiotensin II [39, 40]. Angiotensin II further regulates the renin-angiotensin-aldosterone system (RAAS) and plays a crucial role in response to inflammation [41]. In normal individuals, ACE2 mRNA and protein are highly expressed in the gastrointestinal system, especially in the absorptive enterocytes of the ileum and colon [42]. SARS-CoV-2 spike (S) glycoproteins induce direct cytopathic effects upon interactions with the ACE2 receptors and further downregulates ACE2 expression [43], which in turn renders this enzyme incapable of exerting its protective effects and results in dysregulated RAAS, leading to malabsorption, diarrhea, and other intestinal disorders [40, 44, 45]. Second, SARS-CoV-2 probably regulates tryptophan absorption through intestinal ACE2 to change the gut microbiota, causing intestinal inflammation [45, 46]. Finally, the “cytokine storm” has been an essential mechanism of multiple organ dysfunction involving the digestive system since the COVID-19 outbreak [37, 46]. High expressions of proinflammatory cytokines such as interferon-*γ* (IFN-*γ*), IFN-*α*, and IL-6 were observed in the serum of patients with COVID-19. Monoclonal antibodies targeting the excessive release of inflammatory cytokines may be promising for COVID-19 treatment [23, 24].

Inactivated vaccines have been widely used in China, and several randomized clinical trials have proven these vaccines' safety, tolerability, and immunogenicity [47–49]. The Guangdong pandemic exhibited that the inactivated vaccines could yield overall effectiveness of 59.0% against the Delta variant among the aged 18–59 years [50]. Our study indicated that the inactivated vaccines (Sinovac or Sinopharm) might help prevent severe or critical symptoms and prevent Delta variant-induced liver injury. Moreover, recent studies have indicated that the antibody levels of mRNA vaccines (BNT162b2) decreased on an average to 7% of their peak level at six months after vaccination [51], and similar antibody responses were also observed in inactivated vaccines (Sinovac) at six months after the last immunization [52]. In addition, rapidly and markedly high serological antibody responses were observed following mRNA (BNT162b2) or inactivated (Sinovac) booster vaccines [52–54]. Consistent with our results, it is necessary to get the booster vaccines for whom >6 months had elapsed since the last vaccination to protect people's health.

### 4.1. Limitations

The present study has certain limitations. First, the single-center design of the study may have resulted in a selection bias. Previous research indicates that the quality of life is strongly associated with the GSRS, while age may not correlate with change in the GSRS score [55, 56]. It should be noted that the current study did not rule out the confounding factor of age due to the unique transmission of COVID-19 in Yangzhou; a future study is needed to rule out age bias and identify a clear association between COVID-19 and the GSRS score. Additionally, the recall bias should not be neglected. The present study calculated the total GSRS scores through “ask and answer,” and the clinician's expectations may have introduced a positive bias into our findings. Furthermore, as gastrointestinal bleeding or vomiting could not be measured by the GSRS scores, other biases may exist. The two different inactivated vaccines (Sinovac and Sinopharm) were not compared in the present study due to insufficient information, and updated RUCAM was not applied in this study to define the causality of drug-induced liver injury among COVID-19 patients. Finally, the efficiency of the inactivated vaccines after six months could not be compared because only two patients had received the inactivated vaccines six months earlier.

## 5. Conclusions

In summary, serum AST, DBIL LDH, and IL-6 levels are the potential markers for distinguishing severe or critical patients in the early stage of Delta variant infection. Additionally, changes in the levels of these serum makers are transient, and the levels can return to normal after treatment. Furthermore, severe gastrointestinal discomfort was significantly more prevalent in patients with the severe or critical diseases and should thus be considered in patients diagnosed with Delta variant infection. Finally, inactivated vaccines may prevent severe or critical symptoms and Delta variant-induced liver injury. Moreover, the two-dose inactivated vaccine is highly efficient in preventing severe or critical symptoms. Digestive symptoms appear crucial in the early stage of COVID-19, and vaccination programs must be promoted to protect public health.

## Figures and Tables

**Table 1:** Baseline characteristics of patients infected with Delta variant.

	Total	Mild	Moderate	Serve or critical
	*N* = 208	*N* = 32	*N* = 156	*N* = 20
Age (mean ± SD, years)	53.5 ± 20.9	31.5 ± 18.5	54.7 ± 18.1	78.7 ± 6.7
Sex (N, %)				
Male	90 (44%)	15 (47%)	64 (41%)	11 (55%)
Female	118 (56%)	17 (53%)	92 (59%)	9 (45%)
Common symptoms (N, %)				
Fever	98 (47%)	12 (37.5%)	66 (42%)	20 (100%)
Fatigue	74 (35%)	9 (28%)	52 (33%)	13 (65%)
Dry cough	99 (47%)	14 (43%)	75 (48%)	10 (50%)
Myalgia	58 (27%)	7 (21%)	41 (26%)	10 (50%)
Common digestive system symptoms (N, %)				
Diarrhea	24 (12%)	3 (9%)	17 (11%)	4 (25%)
Nausea	21 (10%)	3 (9%)	15 (10%)	3 (15%)
Vomiting	15 (7%)	2 (6%)	11 (7%)	2 (10%)
Abdominal pain	17 (8%)	3 (9%)	11 (7%)	3 (15%)
Comorbidities (N, %)				
Hypertension	57 (27%)	6 (18%)	40 (25%)	11 (55%)
Diabetes	17 (8%)	0	14 (9%)	3 (15%)
COPD	6 (2%)	0	2 (1%)	4 (20%)
Coronary heart disease	25 (12%)	0	10 (6%)	5 (25%)
Others				
Heart rate (mean ± SD, bpm)	90.9 ± 13.7	96.5 ± 12.8	90.2 ± 13.2	88 ± 16.9
Respiratory rate (mean ± SD, breaths/min)	19.1 ± 1.9	19.1 ± 1.2	18.8 ± 1.5	21.7 ± 3.9

SD: standard deviation; COPD: chronic obstructive pulmonary disease.

**Table 2:** Laboratory findings of patients infected with Delta variant on admission to hospital.

	Normal range	Total	Mild	Moderate	Serve or critical
		*N* = 208	*N* = 32	*N* = 156	*N* = 20
White blood cell count (×10^9^/L, mean ± SD)<	3.5–9.5	5.7 ± 3.1	5.4 ± 1.5	5.5 ± 1.9	7.6 ± 8.0
Lymphocyte count (×10^9^/L, mean ± SD	1.1–3.2	1.2 ± 0.6	1.5 ± 1.2	1.1 ± 0.5	0.9 ± 0.3
Platelet count (×10^9^/L, mean ± SD)	125–350	178.9 ± 65.4	208.7 ± 60.3	177.6 ± 65.8	141.8 ± 49.6
D-dimer (mg/L, mean ± SD)	0–500	0.9 ± 2.4	0.3 ± 0.1	0.7 ± 1.6	3.3 ± 6.1
Creatine kinase (U/L, mean ± SD)	<171	141.6 ± 171.5	105.9 ± 78.1	130.1 ± 152	288.4 ± 309
Creatine kinase–MB (U/L, mean ± SD)	<25	18.5 ± 22.5	17.6 ± 9.2	17.8 ± 22.1	25.4 ± 36.2
Lactate dehydrogenase (U/L, mean ± SD)	100–280	227.6 ± 93	218.9 ± 80.4	199.1 ± 56	399.4 ± 144.8
Alanine aminotransferase (U/L, mean ± SD)<	10–40	26.2 ± 25.2	24.9 ± 27.1	26 ± 26.9	26.2 ± 25.2
Aspartate aminotransferase (U/L, mean ± SD)	8–40	33.1 ± 10.5	29.7 ± 6.1	32.5 ± 8.4	43 ± 21
Total bilirubin (*μ*mol/L, mean ± SD)	3–17	9.6 ± 5.2	7.7 ± 3.2	9.6 ± 5.2	12.7 ± 5.7
Direct bilirubin (*μ*mol/L, mean ± SD	<6	5.7 ± 7.2	3.4 ± 1.1	5.1 ± 4.6	13.8 ± 17.4
Creatinine (*μ*mol/L, mean ± SD)	88–176	73.4 ± 24.9	64.1 ± 17.9	72.3 ± 21.9	96.8 ± 39.9
C-reaction protein (mg/L, mean ± SD)	<8	25.9 ± 35	20.4 ± 26.6	20.9 ± 30.5	76.7 ± 55.5
Interleukin-6 (pg/mL, mean ± SD)	<7	25.9 ± 31.5	12.6 ± 11.7	20.4 ± 22.6	80 ± 52.9

SD: standard deviation.

**Table 3:** Comparing the abnormal ratio of Serum markers among three groups.

Group	ALT	AST	TBIL	DBIL	LDH	IL-6
*N* = abnormal counts	N (%)	N (%)	N (%)	N (%)	N (%)	N (%)
Mild	4 (12.1%)	3 (6.8%)^1,2^	0 (0)^1^	0 (0)^3^	3 (9%)^1,2^	21 (37%)^1,2^
Moderate	26 (78.8%)	33 (75%)^1,2^	13 (72%)	33 (77%)^3^	17 (54%)^1,2^	118 (37%)^1,2^
Serve or critical	3 (9.1%)	8 (18.2%)	5 (27%)	10 (23%)^3^	11 (37%)	11 (37%)
Total	33 (100%)	44 (100%)	18 (100%)	43 (100%)	31 (100%)	159 (100%)
X^2^	0.084	6.83	9.675	18.412	27.883	12.59
P	0.772	0.033	0,008	<0.001	<0.001	0.003

Alanine aminotransferase: ALT; aspartate aminotransferase: AST; total bilirubin: TBIL; direct bilirubin: DBIL; lactate dehydrogenase: LDH; interleukin-6:IL-6 1: compared to the serve or critical group, *P* <  0.05 2: compared to the mild or moderate group, *P*> 0.05 3. Compared to the other group, *P* < 0.05.

**Table 4:** Analysis of variance of total GSRS scores among three groups.

Group	Mean	SD	*P*
Mild	12	1.4	*P* ^1^ < 0.001, *P*^2^ > 0.05
Moderate	12.65	1.08	*P* ^1^ < 0.001, *P*^2^ > 0.05
Sever or critical	19.5	1.04	
F	91.773		
P	<0.001		

P^1^: compared with the serve or critical group P^2^: compared with the mild or moderate group.

**Table 5:** Comparing the ratio of the vaccinated population in three groups.

Group	Vaccinated	Nonvaccinated
Mild (N, %)	20 (17.5%)^1,2^	12 (12.8%)
Moderate (N, %)	89 (78.1%)^1,2^	67 (71.2%)
Sever or critical (N, %)	5 (4.4%)	15 (16%)
Total	114	94
*X * ^2^	8.256	
*P*	0.016	

1: compared to the serve or critical group, *P* <  0.052: compared to the mild or moderate group, *P*> 0.05.

**Table 6:** Comparing the efficiency of single-dose and two-dose inactivated vaccines in three groups.

Group	Single-dose	Two-dose
Mild (N, %)	2(10%)	18 (90%)^1,2^
Moderate (N, %)	27 (30.3%)	62 (69.7%)^1,2^
Sever or critical (N, %)	4 (80%)	1 (20%)
Total	33	81
*X * ^2^	9.91	
*P*	0.007	

1: compared to the serve or critical group, *P* <  0.052: compared to the mild or moderate group,*P* > 0.05.

**Table 7:** Comparing the ratio of the abnormal serum markers in the vaccinated and nonvaccinated cohorts.

Group	ALT	AST	TBIL	DBIL	LDH
Nonvaccinated (N, %)	21 (63.6%)	31 (70.4%)	9 (50%)	25 (58.1%)	18 (58%)
Vaccinated (N, %)	12 (36.4%)	13 (29.6%)	9 (50%)	18 (41.9%)	13 (42%)
Total	33	44	18	43	31
*X * ^2^	5.387	14.378	2.355	3.034	2.437
P	0.02	<0.001	0.125	0.082	0.119
OR	0.409	0.262			

Alanine aminotransferase: ALT; aspartate aminotransferase: AST; total bilirubin: TBIL; direct bilirubin: DBIL; lactate dehydrogenase: LDH; interleukin-6:IL-6; OR: odds ratio.

## Data Availability

The data used to support the conclusions of this article are available from the corresponding authors upon request.

## Supplementary Materials

Supplementary Materials Supplementary File 1: Chinese official criterion of four different subtypes of COVID-19. Supplementary File 2: Chinese version of the gastrointestinal symptom rating scale. Supplementary File 3: the abnormal serum markers ratio on day 7, day 14, and day 21.
