# Supplementary material for: Real-World Evidence for COVID-19 Delta Variant's Effects on the Digestive System and Protection of Inactivated Vaccines from a Medical Center in Yangzhou, China: A Retrospective Observational Study
Source: Int J Clin Pract. 2022 Aug 19;2022:7405448. doi: 10.1155/2022/7405448 (PMC9417746; doi:10.1155/2022/7405448)
Supplement: Supplementary Materials — Supplementary File 1: Chinese official criterion of four different subtypes of COVID-19. Supplementary File 2: Chinese version of the gastrointestinal symptom rating scale. Supplementary File 3: the abnormal serum markers ratio on day 7, day 14, and day 21. [file 7405448.f1.zip › 7405448.f1/Supplementary File 1 Criterion of four different subtypes of Covid-19 in China.docx]

## Criterion of four different subtypes of Covid-19 in China

There are four subtypes of Covid-19 in China: mild, moderate, severe, critical based on the eighth edition of Chinese official guidelines for Covid-19.

**Mild illness:**

Individuals who have slight symptoms that are consistent with Covid-19, and have no abnormal chest imaging.

**Moderate illness:**

Individuals who have fever or show typical evidence of lower respiratory disease, and have typical Covid-19 chest imaging.

**Severe illness:**

Can be diagnosed when meet any following criteria.

For adults:

1. Individuals who present with shortness of breath or respiratory rate (RR) >30 breaths/min.
2. Individuals who have SpO2 <94% on room air at sea level
3. The ratio of arterial oxygen partial pressure to fractional inspired oxygen (PaO2/FiO2) <300 mm Hg.
4. Individuals who clinical symptoms progressive aggravation or lung infiltrates >50% in 48 h.

For children:

1. Individuals who Fever >38.0°C lasting ≥3 days.
2. Individuals who present with shortness of breath exclude the influence of crying or fever (respiratory rate (RR) >60 breaths/min for 0~2 months, RR ≥50 breaths/min for 2~12 months, RR ≥40 breaths/min for 1~5 years old, RR ≥30 breaths/min for ≥5 years old).
3. Individuals who have SpO2 <94% on room air at sea level
4. Individuals who present with three-concave disease or nasal ala flap.
5. Individuals who present with drowsiness or convulsions.
6. Individuals who present with feeding intolerance or dehydration.

**Critical illness:**

Can be diagnosed when meet any following criteria.

1. Individuals who have respiratory failure and need mechanical ventilation.
2. Individuals who present with septic shock.
3. Individuals who present with multiple organ dysfunction.
