# Supplementary material for: Real-World Evidence for COVID-19 Delta Variant's Effects on the Digestive System and Protection of Inactivated Vaccines from a Medical Center in Yangzhou, China: A Retrospective Observational Study
Source: Int J Clin Pract. 2022 Aug 19;2022:7405448. doi: 10.1155/2022/7405448 (PMC9417746; doi:10.1155/2022/7405448)
Supplement: Supplementary Materials — Supplementary File 1: Chinese official criterion of four different subtypes of COVID-19. Supplementary File 2: Chinese version of the gastrointestinal symptom rating scale. Supplementary File 3: the abnormal serum markers ratio on day 7, day 14, and day 21. [file 7405448.f1.zip › 7405448.f1/Supplementary File 2 Chinese version of the gastrointestinal symptom rating scale.docx]

# Chinese version of the gastrointestinal symptom rating scale

***This survey contains questions about how you have been feeling and what it has been like during the past week. Mark the choice that best applies to you and your situation. The information that you provide will remain strictly confidential.***

**1. During the past week, have you had any pain in the upper part of your abdomen or pit of your stomach?**

1. no or temporary discomfort

2. occasional discomfort that lasts for a short time

3. frequent discomfort that affects daily life

4. continuous discomfort that severely affects daily life

**2. During the past week, have you had any discomfort related to reflux? (Sensation of regurgitating small quantities of acid or flow of sour or bitter fluid from the stomach up to the throat.)**

1. no or temporary discomfort

2. occasional discomfort that lasts for a short time

3. frequent discomfort that affects daily life

4. continuous discomfort that severely affects daily life

**3. During the past week, have you felt any burning in your abdomen? (Unpleasant stinging or burning sensation in the chest.)**

1. no or temporary discomfort

2. occasional discomfort that lasts for a short time

3. frequent discomfort that affects daily life

4. continuous discomfort that severely affects daily life

**4. During the past week, have you had any pain due to hunger? (Hollow feeling in the stomach associated with the need to eat between meals.)**

1. no or temporary discomfort

2. occasional discomfort that lasts for a short time

3. frequent discomfort that affects daily life

4. continuous discomfort that severely affects daily life

**5. During the past week, have you suffered from nausea? (Feeling of wanting to throw up or vomit.)**

1. no or temporary discomfort

2. occasional discomfort that lasts for a short time

3. frequent discomfort that affects daily life

4. continuous discomfort that severely affects daily life

**6. During the past week, have you felt any gurgling in your stomach? (Vibrations or noise in the stomach.)**

1. no or temporary discomfort

2. occasional discomfort that lasts for a short time

3. frequent discomfort that affects daily life

4. continuous discomfort that severely affects daily life

**7. During the past week, have you had any stomach bloating? (Swelling often associated with sensation of gas or air in the stomach.)**

1. no or temporary discomfort

2. occasional discomfort that lasts for a short time

3. frequent discomfort that affects daily life

4. continuous discomfort that severely affects daily life

4. continuous discomfort that severely affects daily life

**8. During the past week, have you had any problems with eructation? (Bringing up air or gas from the stomach via the mouth.)**

1. no or temporary discomfort

2. occasional discomfort that lasts for a short time

3. frequent discomfort that affects daily life

4. continuous discomfort that severely affects daily life

4. continuous discomfort that severely affects daily life

**9. During the past week, have you had any need for farting? (Need to release air or gas from the bowel.)**

1. no or temporary discomfort

2. occasional discomfort that lasts for a short time

3. frequent discomfort that affects daily life

4. continuous discomfort that severely affects daily life

**10. During the past week, have you suffered from constipation? (Reduced ability to empty the bowels.)**

1. no or temporary discomfort

2. occasional discomfort that lasts for a short time

3. frequent discomfort that affects daily life

4. continuous discomfort that severely affects daily life

**11. During the past week, have you been bothered by diarrhea? (Too frequent emptying of the bowels.)**

1. no or temporary discomfort

2. occasional discomfort that lasts for a short time

3. frequent discomfort that affects daily life

4. continuous discomfort that severely affects daily life

**12. During the past week, has there been any softening in your stool?**

1. no or temporary discomfort

2. occasional discomfort that lasts for a short time

3. frequent discomfort that affects daily life

4. continuous discomfort that severely affects daily life

**13. During the past week, has there been any hardening in your stool?**

1. no or temporary discomfort

2. occasional discomfort that lasts for a short time

3. frequent discomfort that affects daily life

4. continuous discomfort that severely affects daily life

**14. During the past week, have you had the any sudden urges for stool? (Urgent need to go to the toilet associated with a feeling that you are not in full control.)**

1. no or temporary discomfort

2. occasional discomfort that lasts for a short time

3. frequent discomfort that affects daily life

4. continuous discomfort that severely affects daily life

**15. When going to the toilet during the past week, have you had the sensation of not completely emptying the bowels? (Still feel a need to pass more stool despite having exerted yourself to do so.)**

1. no or temporary discomfort

2. occasional discomfort that lasts for a short time

3. frequent discomfort that affects daily life

4. continuous discomfort that severely affects daily life
