# Supplementary material for: Real-World Evidence for COVID-19 Delta Variant's Effects on the Digestive System and Protection of Inactivated Vaccines from a Medical Center in Yangzhou, China: A Retrospective Observational Study
Source: Int J Clin Pract. 2022 Aug 19;2022:7405448. doi: 10.1155/2022/7405448 (PMC9417746; doi:10.1155/2022/7405448)
Supplement: Supplementary Materials — Supplementary File 1: Chinese official criterion of four different subtypes of COVID-19. Supplementary File 2: Chinese version of the gastrointestinal symptom rating scale. Supplementary File 3: the abnormal serum markers ratio on day 7, day 14, and day 21. [file 7405448.f1.zip › 7405448.f1/Supplementary File 3 The abnormal serum markers ratio on day 7, day 14, and day 21.docx]

## The abnormal serum markers ratio on day 7, day 14, and day 21

**Day 7 and Day 14**

**ALT**

|  | Day 7 | Day 14 | Total |
| --- | --- | --- | --- |
| **Normal** | 23 (43.4%) | 30 (56.6%） | 53 |
| **Abnormal** | 10 (76.9%) | 3 (23.1%) | 13 |
| **X^2^** | 4.694 | | |
| **P** | 0.03 | | |

**AST**

|  | Day 7 | Day 14 | Total |
| --- | --- | --- | --- |
| **Normal** | 20 (33.9%) | 39 (66.1%) | 59 |
| **Abnormal** | 24 (82.5%) | 5 (17.5%) | 29 |
| **X^2^** | 4.694 | | |
| **P** | 0.03 | | |

**Tbil**

|  | Day 7 | Day 14 | Total |
| --- | --- | --- | --- |
| **Normal** | 16 (47.1%) | 18 (52.9%) | 34 |
| **Abnormal** | 2 (100%) | 0 | 2 |
| **X^2^** | 2.89 | | |
| **P** | 0.089 >0.05 | | |

**Dbil**

|  | Day 7 | Day 14 | Total |
| --- | --- | --- | --- |
| **Normal** | 30 (47.1%) | 39 (52.9%) | 69 |
| **Abnormal** | 13 (100%) | 4 | 17 |
| **X^2^** | 5.939 | | |
| **P** | 0.015 | | |

**LDH**

|  | Day 7 | Day 14 | Total |
| --- | --- | --- | --- |
| **Normal** | 24 (46.2%) | 28 (53.8%) | 52 |
| **Abnormal** | 7 (70%) | 3 (30%) | 10 |
| **X^2^** | 1.908 | | |
| **P** | 0.167 | | |

**Day 14 and Day 21**

**ALT**

|  | Day 14 | Day 21 | Total |
| --- | --- | --- | --- |
| **Normal** | 30 (47.6%) | 33 (52.4%） | 63 |
| **Abnormal** | 3(100%) | 0 | 3 |
| **X^2^** | 1.397 | | |
| **P** | 0.237 | | |

**AST**

|  | Day 14 | Day 21 | Total |
| --- | --- | --- | --- |
| **Normal** | 39 (48.1%) | 42 (51.9%） | 81 |
| **Abnormal** | 5(71.4%) | 2 (28.6%) | 7 |
| **X^2^** | 1.439 | | |
| **P** | 0.23 | | |

**Dbil**

|  | Day 14 | Day 21 | Total |
| --- | --- | --- | --- |
| **Normal** | 39 (47.5%) | 43(52.5%） | 82 |
| **Abnormal** | 4(80%) | 1 (20%) | 5 |
| **X^2^** | / | | |
| **P** | 0.116 | | |

|  | Day 14 | Day 21 | Total |
| --- | --- | --- | --- |
| **Normal** | 28 (47.5%) | 31(52.5%） | 59 |
| **Abnormal** | 3(100%) | 0 (20%) | 5 |
| **X^2^** | 1.401 | | |
| **P** | 0.237 | | |

**LDH**
